# Analysis of Hot Stamping Tool Cooling System—A Case Study

**DOI:** 10.3390/ma14112759

**Published:** 2021-05-23

**Authors:** Piotr Danielczyk, Ireneusz Wróbel

**Affiliations:** Faculty of Mechanical Engineering and Computer Science, University of Bielsko-Biala, Willowa 2, 43-309 Bielsko-Biala, Poland; iwrobel@ath.bielsko.pl

**Keywords:** hot stamping, cooling channels, stamping analysis, CFD analysis

## Abstract

One of the important steps in the design of hot stamping tools is the analysis of their cooling system. This article presents an authorial, two-stage approach to solving this problem. The first stage consisted of a series of simulations of the hot stamping process in the Autoform package, with initial selection of shape and arrangement of cooling channels. These results allowed for the design of the tool for which the coupled thermal-flow analysis was carried out. The correctness of the adopted design assumptions has been confirmed by experimental tests. A trial series of drawpieces made in production conditions meet the requirements for hardness, mechanical properties, and appropriate microstructure. The presented procedure has become the practice of the drawpiece producers.

## 1. Introduction

The modern automotive industry faces major challenges in terms of sustainable development [1]. They mainly concern the reduction of CO_2_ emissions, which is directly related to energy saving and the efficient use of natural resources. These activities should have a direct impact on the reduction of exhaust emissions from vehicles with traditional drive systems and stimulate the rapid development of electric vehicles [2]. For this reason, the goal is to reduce the weight of passenger car bodies while meeting all safety standards. It can be noticed that this trend has been constant for over a dozen years [3]. New car models are lighter than their predecessors by at least 20% [4], and in electric cars, the reduction in body weight to some extent compensates for the weight gain resulting from the use of batteries. Therefore, the production of body elements of modern passenger cars requires the use of new construction materials and production technologies. As a result, more and more body parts of new car models are produced by hot-stamping which enables the production of high-strength drawpieces with relatively low weight [5]. After the hot stamping process, the material of a drawpiece can achieve a tensile strength of more than 1300 MPa. In addition, this technology enables the production of drawpieces with zones of different thicknesses, and thus with different mechanical properties in these zones, which in cold stamping process is extremely difficult. During hot stamping, a drawpiece is shaped and hardened at the same time [6]. The material from ferritic-pearlitic structure is transformed into a martensitic structure. In order to achieve this effect, a high cooling rate of at least 27 °K/s is essential [7]. This requires the production of a special tool with appropriately selected cooling channels [8].

The process of designing the tool begins with computer simulations of the hot stamping process. Based on a drawpiece CAD model, all tool elements are built, and computer simulations of the process are performed using the finite element method. The results of FLD (Forming Limit Diagram) [9] are analyzed. Hardening deformations [10,11] and the results related to the hardening process, such as hardness and the percentage of martensite in the drawpiece are assessed. The result of this analysis is a set of CAD models of tools whose shape (obtained by computer simulation) enables the production of drawpieces with the assumed accuracy. The next important stage is the analysis of the tool cooling process whose results make it possible to select the arrangement and diameters of the cooling channels in order to achieve the required temperature of the tool surface in the final stage of the process. It is assumed that the maximum temperature on the surface of the tool can then be at most 200 °C. If areas where the temperature exceeds the permissible values are detected, the diameters of some channels and their position in relation to the working surfaces of the tool are changed, so as not to exceed the required 200 °C. The result of this analysis is the basis for the development of the tool design. When the tool and trial series of drawpieces are made, it is necessary to check the parameters related to both dimensional accuracy and expected mechanical properties [12,13,14].

In the literature on problems related to the analysis of cooling of hot stamping tools, much attention is paid to tools made in additive technologies, with cooling channels of a complicated shape, the so-called conformal channels. Such tools have many advantages over those with drilled channels. They have, first, a more even temperature distribution on the tool surface and the lack of thermal bridges has a positive effect on their wear. This problem is discussed in works [15,16,17] which highlight a significant improvement in cooling conditions for molds made in additive technologies. This translates into the quality of the drawpieces. The work [18] showed that the use of molds with conformal channels reduces the production cycle time by up to 25%. A hybrid method of constructing mold cooling channels, which is a combination of additive and milling methods, is described in [19]. New possibilities are provided using high-speed additive manufacturing technology, in particular highly efficient sintering technology for metals. According to the authors in [20], the use of these technologies can accelerate printing and reduce its costs, without losing the accuracy and desired material properties. An interesting method of producing molds with conformal channels with optimal arrangement is described in [21]. The finished mold was made by casting using ceramic cores.

Nevertheless, due to the still high and unacceptable costs of making such tools, especially for large-sized drawpieces, these technologies are not currently used in production conditions. Few works have been devoted to the analysis of cooling systems for tools with drilled channels. These studies most often describe the hot stamping process by means of thermomechanical analyses without taking into account the flow of the cooling medium. Work [22] presents a simulation of the hot stamping process for an exemplary drawpiece. The analysis takes into account the thermal phenomena occurring at the contact of the blank and tools as well as those related to the heat transfer between the cooling channels and the tool. Similar analyses were shown in works [23,24,25,26,27], with attention paid to the methodology of optimal selection of dimensions and location of cooling channels with the use of genetic algorithms and the parameters of the process itself using the Taguchi method. Paper [28] presents the simulation results of the hot forming process together with the analysis of the flow of the cooling medium. The temperature distribution on the surface of the drawpiece and the hardness at selected points obtained as a result of computer simulations were assessed, but no experiments were conducted to confirm the correctness of the obtained results.

For this reason, in the presented work, the process of selecting cooling channels and the analysis of the efficiency of the cooling system for an exemplary tool, based on the results of computer simulations, will be discussed in detail. In addition, the results of hardness tests, mechanical properties, and the evaluation of the microstructure will be shown for the trial series of drawpieces, confirming the correctness of the tool design.

## 2. Object of Analysis

**Figure 1:**
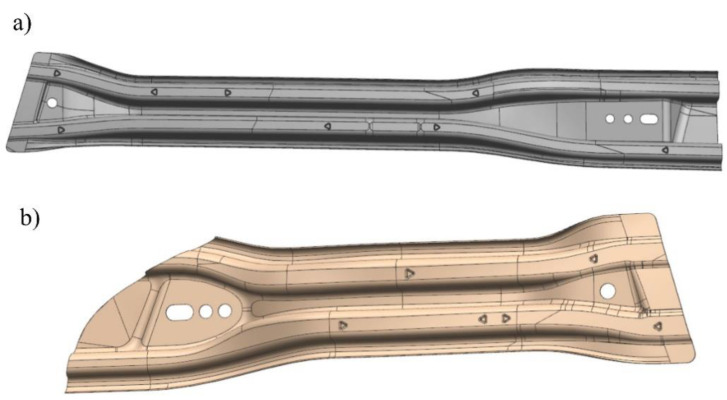
CAD models of door beams. (**a**) long beam; (**b**) short beam.

The object of the analysis is a tool for the production of drawpieces for reinforcing beams mounted in the front (long beam, Figure 1a) and rear (short beam, Figure 1b) door of a passenger car.

These beams are produced in one tool, in left and right variants. They are made of 1 mm, 22MnB5 steel [29,30] intended for hot stamping where a drawpiece is shaped and hardened at the same time. It is assumed that after the pressing process, the drawpiece will have the following mechanical parameters:

- yield point

Rp0.2 = 950–1200 Mpa,(1)

- strenght limit

Rm = 1300–1650 MPa,(2)

- hardness

HV = 400–550.(3)

It is assumed that after the pressing process, the drawpiece will have the following mechanical parameters: yield point Rp0.2 = 950–1200 MPa, strength limit Rm = 1300–1650 MPa, and hardness HV = 400–550. It should be remembered that a correctly designed drawpiece cooling system, ensuring appropriate cooling rate, is one of the most significant factors ensuring the production of a drawpiece with proper mechanical parameters and the desired metallographic structure.

## 3. Choice of Cooling System Computer Simulations

The analysis of the cooling system has been performed in two steps. The first one used the functionality of the Autoform 7.0 hot stamping process simulation software (AutoForm Engineering GmbH, Pfäffikon, Switzerland) [31], which allows for an approximate but very quick analysis of the tool cooling process. This makes it possible to pre-select the arrangement and diameters of cooling channels. The result of this analysis is the basis for the development of the tool design. In the second stage, with a 3D model of the tool, cooling simulations are performed using coupled thermal-flow simulations.

### 3.1. Initial Selection of Cooling System Parameters

**Figure 2:**
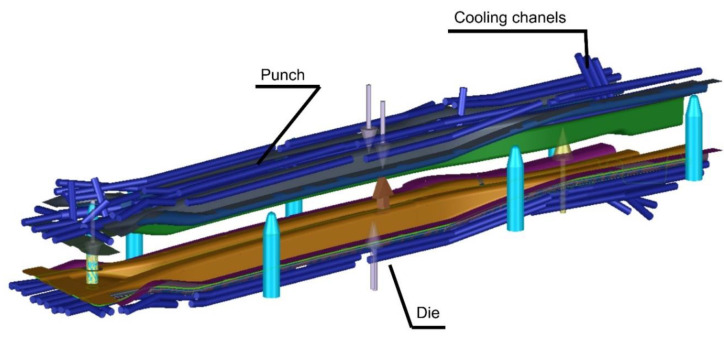
Arrangement of cooling channels in the tool for producing a long beam.

As already noted, the tool cooling analyses in the Autoform system are approximate thermomechanical analyses. In this case, there is no complex and time-consuming CFD (Computational Fluid Dynamics) analysis, only a thermomechanical analysis until reaching a steady state. An appropriate CAD model was prepared for each beam for the die and punch cooling analysis. This model includes working surfaces of the tools, the shape of the blank, center lines of the planned cooling channels appropriately arranged in relation to the working surfaces of the tools and their diameters. Figure 2 shows the arrangement of the cooling channels for the upper and lower part of the tool for one of the long beams. Similar models have been developed for the short beam.

**Figure 3:**
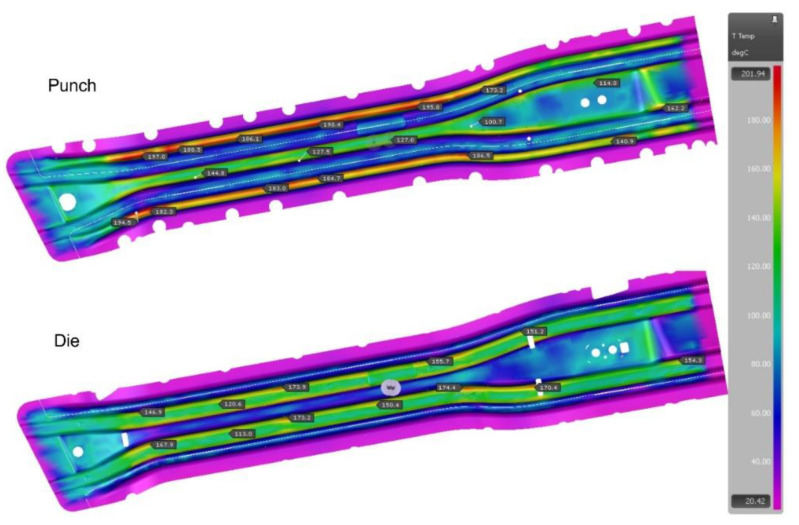
Temperatures of working surfaces of the upper and lower parts of the tool for the long beam.

**Figure 4:**
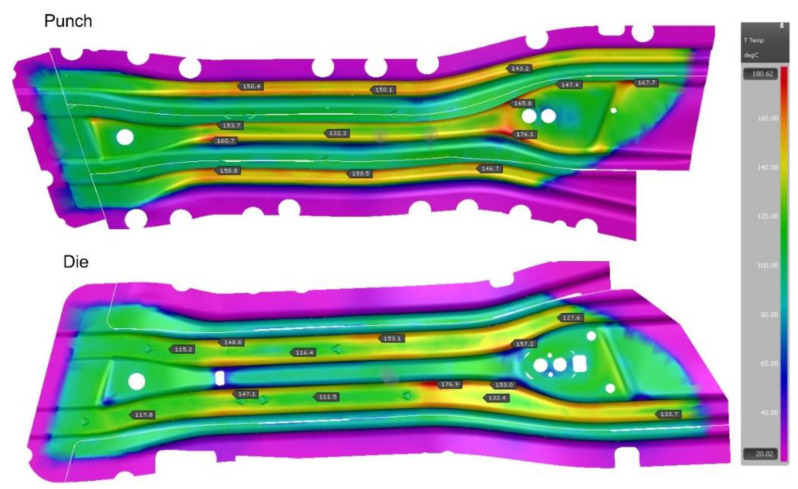
Temperatures of working surfaces of the upper and lower parts of the tool for the short beam.

The analysis of the tool cooling process in the Autoform system is carried out simultaneously with the analysis of the drawpiece pressing and hardening. Preparing such a simulation requires defining the parameters of the stamping process itself (blank temperature, tool temperature, press pressure, hardening time) and providing the value of the heat transfer coefficient from the tool to the cooling medium (water) and the temperature of the cooling medium. This simulation is iterative, and the criterion of convergence of the computational process is the state where the maximum tool temperature differences in subsequent iterations will not exceed 2 °C. A series of simulations of tool heating and cooling process were performed. However, if areas where the temperature on the working surfaces of the tools exceeded the permissible value of 200 °C were noted, diameters of some channels and their position in relation to the tool working surfaces were changed. Figure 3 and Figure 4 show the temperature of the working surfaces of the upper and lower parts of the tool for both beams obtained by simulation, for the best version. It is worth adding that achieving thermal stability required 12 iterations for the long beam and 5 iterations for the short beam.

When analyzing the results presented in the above figures, it can be seen that the temperature of the tool working surfaces did not exceed 200 °C. Most of the surfaces have a much lower temperature, within the range of 120–150°C. Therefore, it can be concluded that the initial arrangement of the cooling channels and their selected diameters ensure the required cooling conditions of the mold. 

### 3.2. Coupled Thermal-Flow Simulations

**Figure 5:**
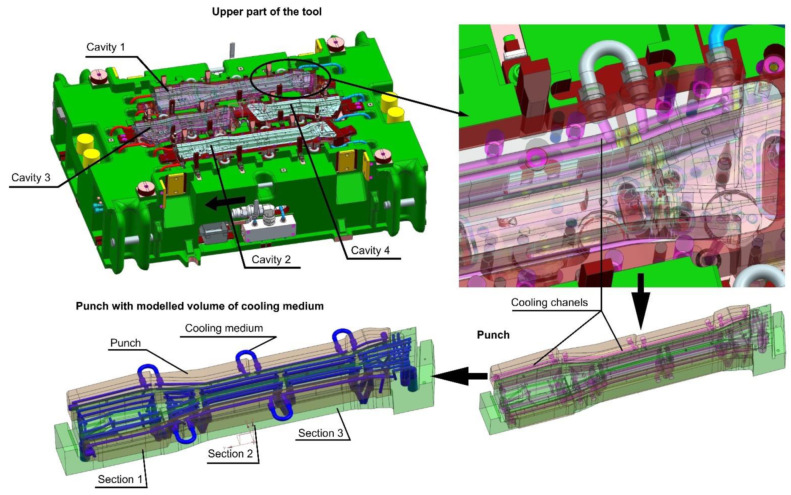
3D CAD model of the upper part of the tool.

The result of the thermomechanical analyses carried out in the Autoform system was the pre-selected and verified shape of the cooling channels along with their diameters. Based on the results of this analysis, three-dimensional models of punches and dies with modelled cooling channels were developed. Figure 5 shows a geometric model of the lower part of the tool with four cavities for the production of a long beam and a short beam in the left and right variants. It is worth paying attention to the construction of the tools themselves. They consist of independent sections (e.g., three for the punch) connected to one another by an additional cooling channel. Dividing the tool into sections gives more freedom in shaping of the cooling channels in the tool. In addition, in case of the tool failure, it is possible to replace a single section, which significantly reduces the costs of regeneration.

**Figure 6:**
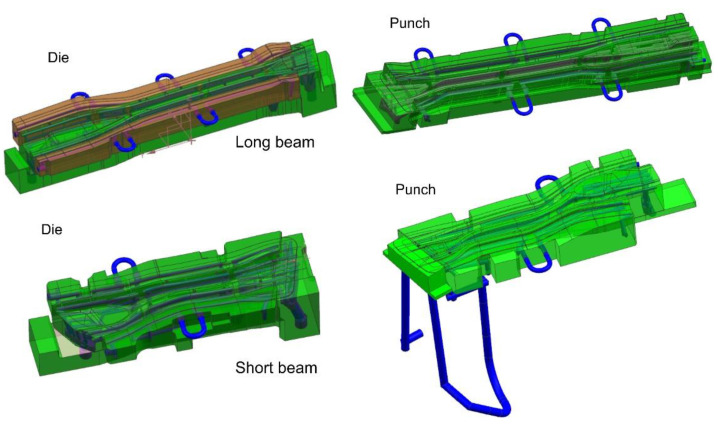
3D CAD models of die and punch prepared for the purpose of thermal-flow analyses.

With a 3D model of the tool, it is possible to prepare coupled thermal-flow simulations. For this purpose, in the next step, die and punch models were developed, along with volumetric models of the cooling medium (Figure 6).

#### 3.2.1. Discrete Calculation Model

**Figure 7:**
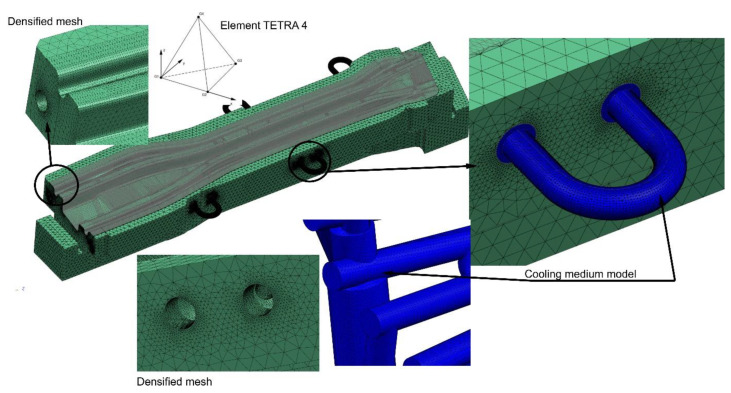
Discrete model of the long beam die.

Based on the developed die and punch 3D models, together with models of the cooling medium, their discrete models were prepared. Figure 7 shows, for example, the discrete model of the long beam die. The mechanical and thermal properties of steel were assigned to the elements modelling the die, and the flow and thermal properties of water were assigned to the discrete model of the cooling medium. Expecting long analysis times and taking into account the complexity of the geometry of the form cooling channels, four-node TETRA4 elements, usually used in such analyses, were used to build the finite element mesh. The discrete die model and the discrete cooling medium model were built with 8 mm elements. Moreover, the FEM mesh was densified (to the size of 1 mm) in places where the drawpiece adhered to the die, and in the boundary layer of the cooling medium. The FEM mesh size for the fluid model was 1 mm. It should be emphasized that the above-mentioned sizes of finite elements in the discrete model were selected in an iterative manner. In subsequent iterations, the size of the finite elements was reduced. The influence of this change on the obtained results (temperature, flow velocity and pressure) was examined. If the results did not change significantly in the consecutive iteration (more than 3%), it was assumed that the finite element sizes from the previous iteration were optimal.

**Figure 8:**
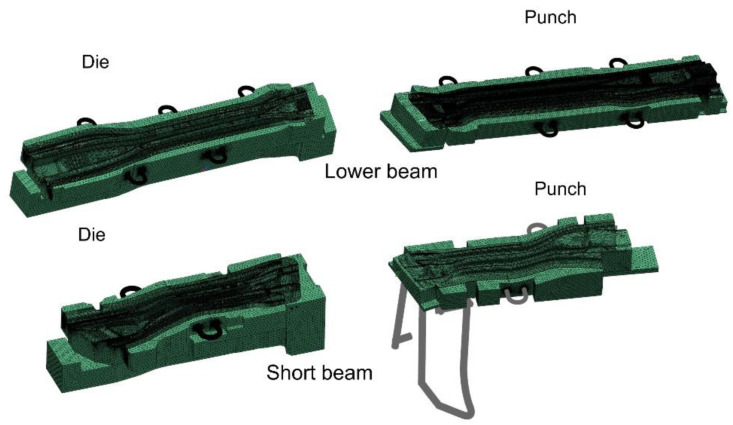
Discrete models of dies and punches for thermal-flow analyses.

The remaining models of dies and punches were developed in a similar way (Figure 8).

The long beam die model consisted of over 8.5 million finite elements and over 1.2 million nodes. The long beam punch model consisted of over 7.5 million elements and over 2 million nodes. On the other hand, the short beam die model consisted of over 4.8 million finite elements and over 1.5 million nodes, and the long beam punch model was over 5 million finite elements and 1.5 million nodes.

#### 3.2.2. Boundary Conditions of the Analysis

The coupled thermal-flow analysis does not include the simulation of stamping process. Therefore, it is necessary to determine the heat flux that the beam gives off to the upper and lower parts of the tool during the forming process. In the process of forming and hardening of the drawpiece, its temperature drops from the initial temperature $t_{2}$ (before the forming process) of 760 °C to the final temperature $t_{1}$ of about 150 °C (after the hardening process). Knowing the forming and hardening time T of 8 s, the mass of the drawpiece m, and the specific heat of the steel c, one can calculate the required heat flux P:

(4) $$P=\frac{c\cdot m\left(t_{2}-t_{1}\right)}{T}$$

**Figure 9:**
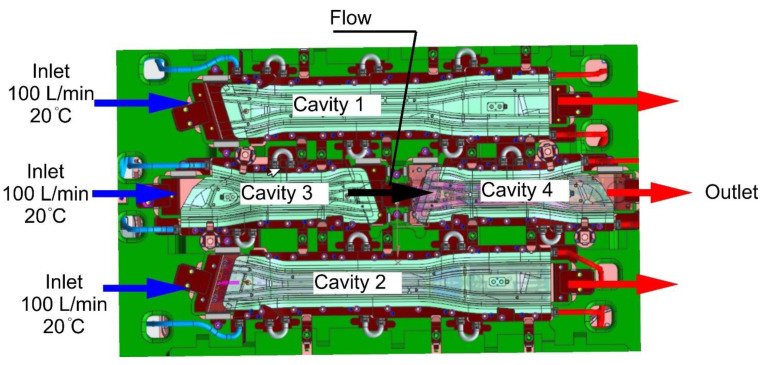
The method of supplying the punch and die with cooling water.

For the long beam, the heat flux is *P* = 54 kW, and for the short beam it is *P* = 23 kW. In addition to the boundary conditions related to the heat exchange between the blank and the tool surfaces, the model also takes into account the conditions for the flow of the cooling medium. In accordance with the preliminary design of the cooling system and the parameters of the medium cooling tower, it was assumed that the system on the inlets side was supplied with a cooling medium with the temperature of 20 °C and the flow of 100 L/min. The open channels were installed on the outlet side of the medium (Figure 9). The method of cooling the mold requires some comment. Cavities 1 and 2 of the tools are supplied independently of each other, which means that both are supplied with water at a flow of 100 L/min and the temperature of 20 °C. Cavity 3 is supplied with water with the temperature of 20 °C and a flow of 100 L/min, while cavity 4 is supplied with water at a temperature and flow velocity resulting from the conditions of flow and cooling of cavity 3.

**Figure 10:**
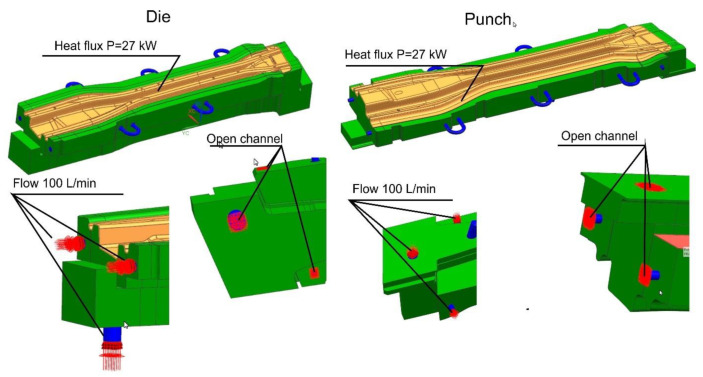
Boundary conditions for the die and the long beam punch model.

Figure 10 shows the boundary conditions defined in the die and the long beam punch model. Models of dies and punches for the remaining beams were prepared in a similar way.

Four calculation models prepared in this way, with the boundary conditions described above, were analyzed with the Simcenter software (Flow module) (version 12, Siemens PLM Software, Plano, TX, USA). Expecting long calculation times, the Fixed Turbulent Viscosity model was adopted, which is a reasonable compromise between the expected accuracy and numerical efficiency [32]. The analysis was performed as steady state, with the convergence criteria assumed in accordance with the default solver settings.

## 4. Results of Computer Simulations

From the point of view of assessing the effectiveness of the proposed cooling system, the temperature of the working surfaces of the tools, as well as the water flow velocity in the cooling channels and the temperature of the cooling medium have been assessed.

### 4.1. Cavity 1—Right Long Beam

**Figure 11:**
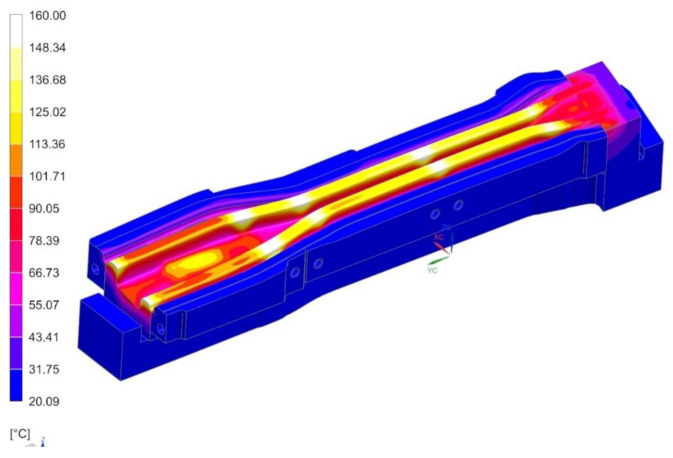
Temperature distribution in the die, °C.

**Figure 12:**
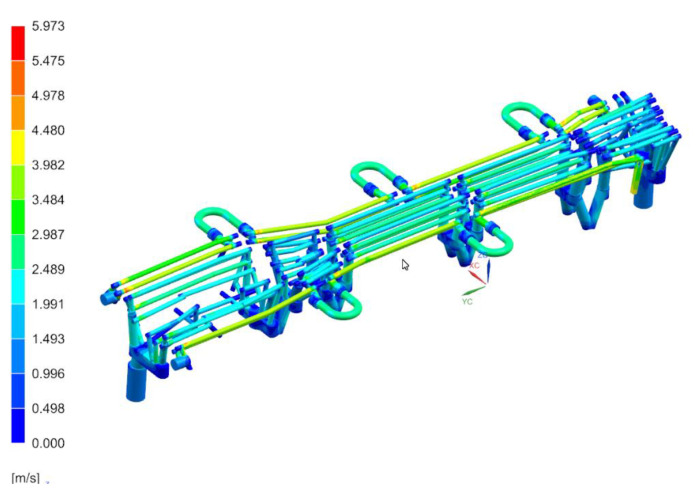
Water flow velocities through the die cooling channels, m/s.

**Figure 13:**
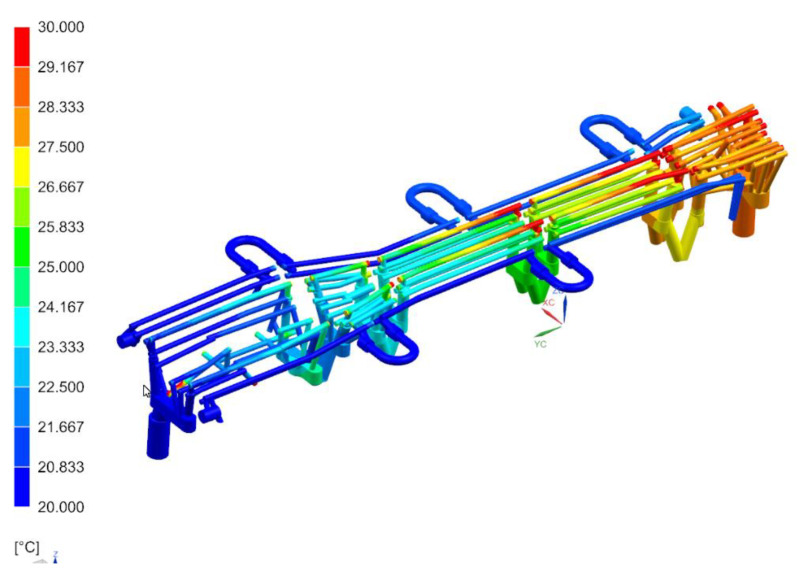
Temperature distribution in the die cooling channels, °C.

Figure 11, Figure 12 and Figure 13 show the temperature distribution in the long beam die, the water flow velocity in the die cooling channels, and the temperature of the cooling medium.

**Figure 14:**
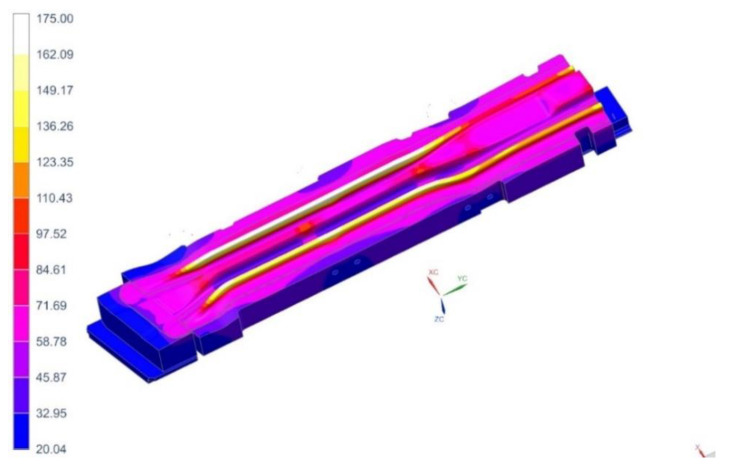
Temperature distribution in the punch, °C.

When analyzing the results obtained for the die, one can conclude that the maximum temperature of the tool working surfaces did not exceed 160 °C, which compared to the limit temperature (200 °C) means that the cooling system meets the requirements of the hot stamping technology. Moreover, in all cooling channels there is a cooling medium flowing, there are no blind channels or those with low flow velocities. The water flow velocity in the cooling channels is above 1 m/s. The temperature of the cooling medium increased from 20 °C at the inlet to a temperature of nearly 28 °C at the outlet. Also, importantly, the flow between the tool sections is not disturbed. Slightly higher temperatures can be observed only in the tool sections joints. However, they do not exceed 30 °C. The temperature distribution on the surface of the long beam punch looks slightly different (Figure 14). The temperature values on the punch surface are higher than in the die, and their maximum value is about 175 °C. For the proposed cooling system, however, the temperature does not exceed the recommended 200 °C. The conclusions regarding the flow of the medium in the punch channels are similar to those presented in the die analysis.

The results of the flow analyses for cavity 2 (long left beam), due to the same analysis conditions, are similar to those described above.

### 4.2. Cavity 3—Short Right Beam

**Figure 15:**
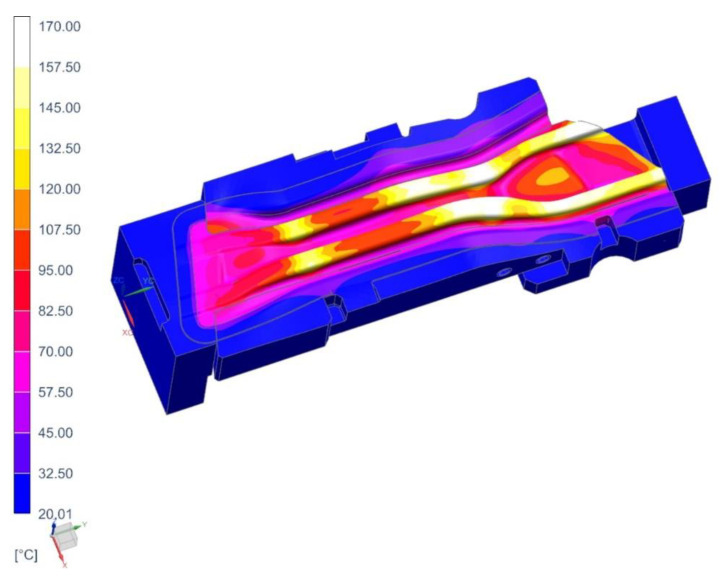
Temperature distribution in the die of cavity 3, °C.

**Figure 16:**
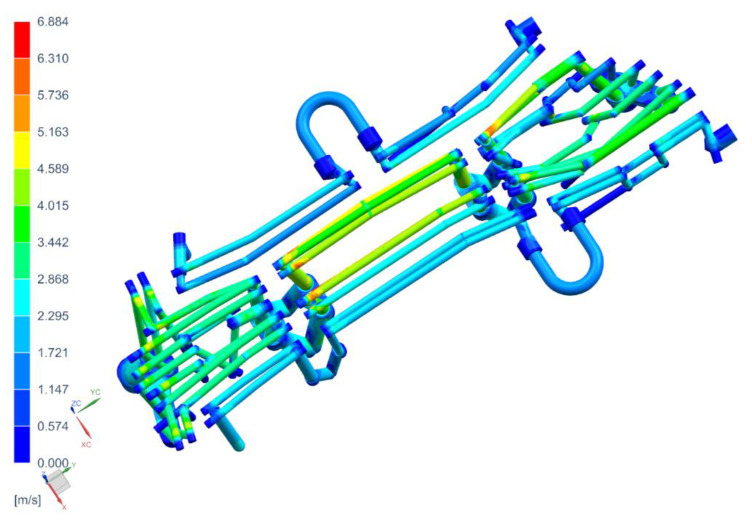
Flow velocity of the cooling medium going through the die of cavity 3, m/s.

**Figure 17:**
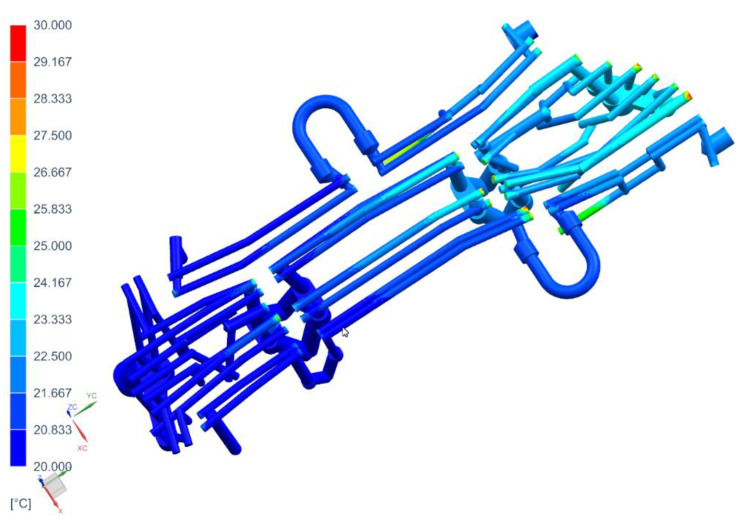
Temperature distribution in the cooling channels of the die of cavity 3, °C.

The following Figure 15, Figure 16 and Figure 17 show the temperature distribution in the short beam die, the water flow velocity in the die cooling channels and the temperature of the cooling medium.

**Figure 18:**
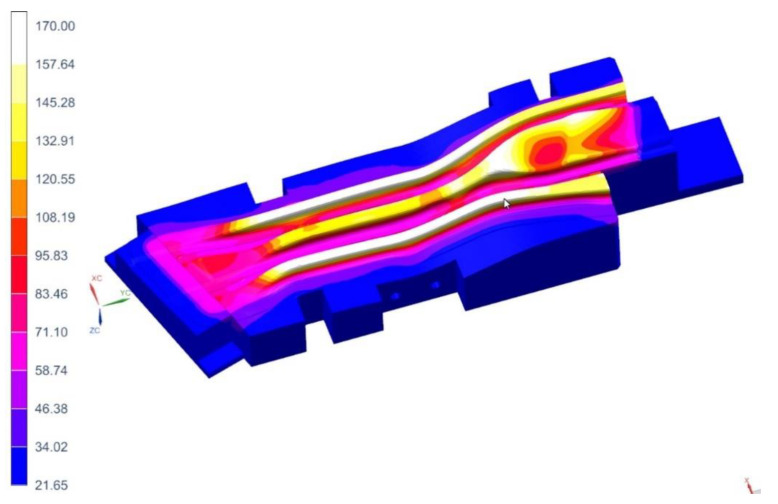
Temperature distribution in cavity 3 punch, °C.

As in the case of the tool for producing the long beam, the maximum temperature of the working surfaces of the tool did not exceed the limit temperature of 200 °C (it was 170 °C). The cooling medium flows in all cooling channels, there are no blind channels. or those with low flow velocities (<1 m/s). The cooling medium temperature rose from 20 °C at the inlet to almost 24 °C at the outlet. As was the case in previous analyses, areas of increased temperature (about 30 °C) can be observed in places where the continuity of the cooling channels is broken. The temperature on the surface of the punch of cavity 3 (Figure 18) does not exceed 128 °C. The conclusions regarding the flow of the medium in the punch channels are similar to those presented in the die analysis.

It is worth adding here that in the die and punch of cavity 4, the temperature values on their surfaces do not exceed 170 °C, and the cooling medium flow conditions are not disturbed.

## 5. Measuring the Temperature of the Tool Working Surfaces with a Thermovision Camera. Measurement of the Drawpiece Properties

**Figure 19:**
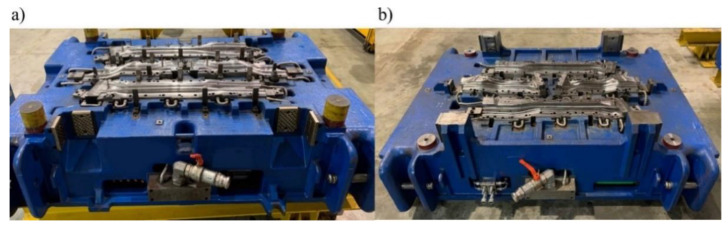
Tool for the production of door beams (**a**) die (**b**) punches.

**Figure 20:**
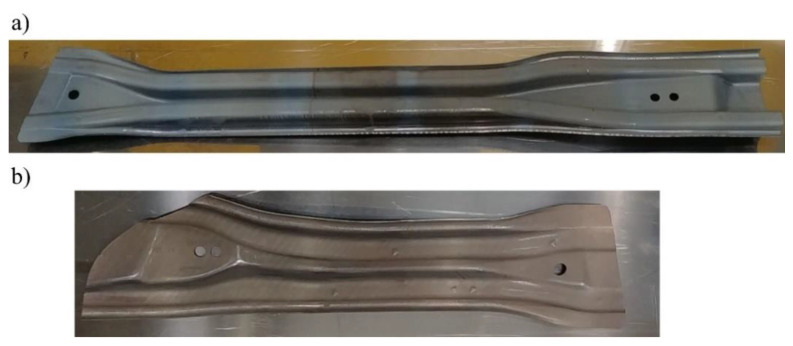
Drawpieces for door beams (**a**) long, (**b**) short.

After performing all the necessary analyses, the tool for the production of door beams was developed (Figure 19). Next, a trial series of drawpieces was made (Figure 20).

### 5.1. Temperature Measurement of Dies and Punches

**Figure 21:**
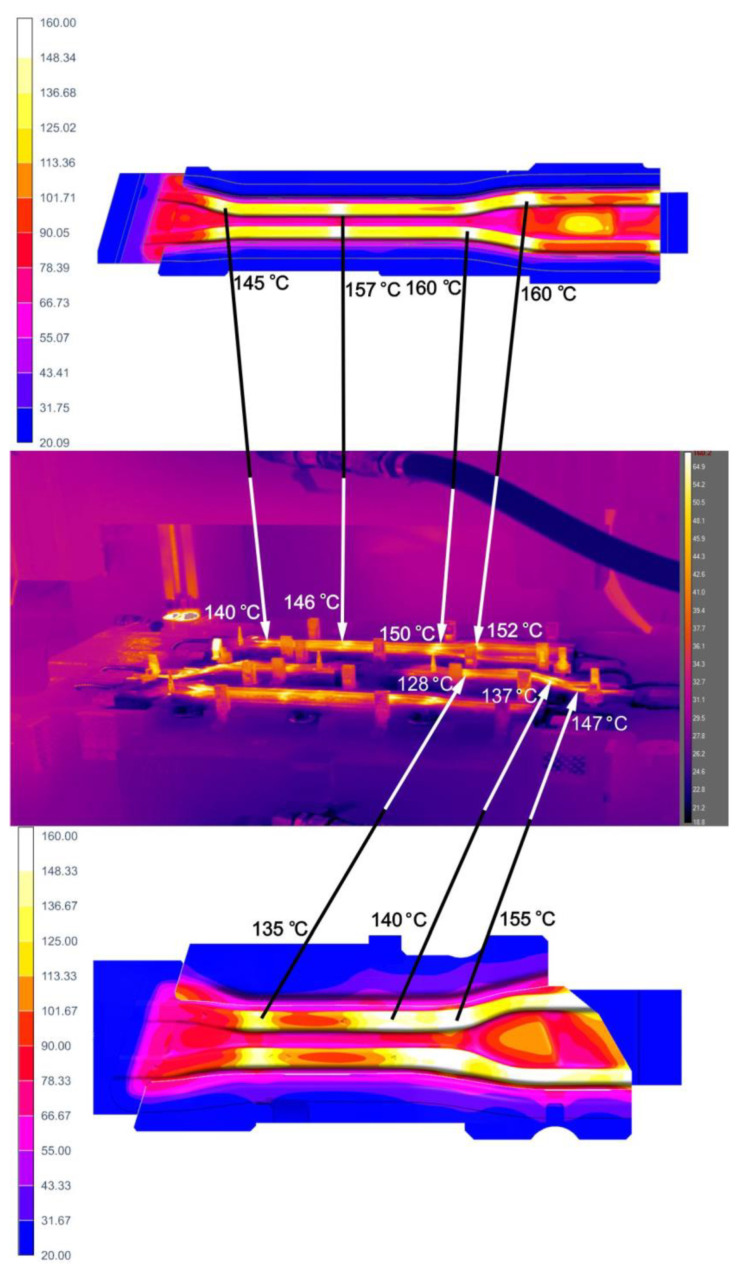
Thermovision measurement of the die for pressing long and short beams, together with the simulation results.

During the production of prototype drawpieces, the temperature of dies and stamps was measured with a Flir A655SC thermovision camera, properly calibrated for the measurement. The procedure took place in production conditions, immediately after the mold was opened and the product was picked up by the robot arm. Figure 21 shows an example of the measurement result of a die for pressing long and short beams performed with a thermovision camera together with the results of analyses obtained by computer simulations.

A detailed analysis of the temperature measurement results on the working surfaces of dies and punches allows us to conclude that:

- Temperature distributions from thermovision camera confirmed the location of the die areas with increased temperatures, obtained by simulation, the maximum values of these temperatures are similar.
- For the long beam die (cavity 1), the temperature measurement result in the place where it had the highest value was 150 °C and the simulation result in the same place was approximately 160 °C.
- For the short beam die (cavity 3), the temperature measurement result in the place where it had the highest value was 147 °C and the simulation result in the same place was approximately 155 °C.

### 5.2. Non-Destructive Tests of Drawpieces

For the trial series of drawpieces, hardness measurements and strength tests were carried out at selected 10 points on the surface of the drawpieces, using a properly calibrated 3MA non-destructive testing device, manufactured by Franchoufer Institution [33].

As a result of the conducted measurements, the following was obtained:

- Vickers hardness:
  ○ for the long beam, the maximum value is 546 (point 5), the minimum value is 474 (point 3),
  ○ o for the short beam, the maximum value is 532 (point 4), the minimum value is 452 (point 2),
- the yield point Rp0.2
  ○ o for the long beam, the maximum value is 1151 MPa (point 8), the minimum value is 984 MPa (point 9),
  ○ o for the short beam, the maximum value is 1151 MPa (point 9), the minimum value is 952 MPa (point 4),
- strength limit Rm
  ○ o for the long beam, the maximum value is 1559 MPa (point 8), the minimum value is 1372 MPa (point 1),
  ○ o for the short beam, the maximum value is 1546 MPa (point 9), the minimum value is 1371 MPa (point 4).

These values are within the assumed range (see Section 2).

In addition, the thickness of the finished drawpiece was checked. Such a measurement in production conditions is made with the help of a thickness gauge at several dozen measuring points; in particular, places are selected where the risk of thinning/thickening of the drawpiece was observed during the simulation of the stamping process. For the discussed beams, the measured values ranged from 0.92 to 1.08 mm, which means that the thinnings and thickenings did not exceed 8% of the nominal thickness of the drawpiece.

### 5.3. Destructive Tests of Drawpieces

**Figure 22:**
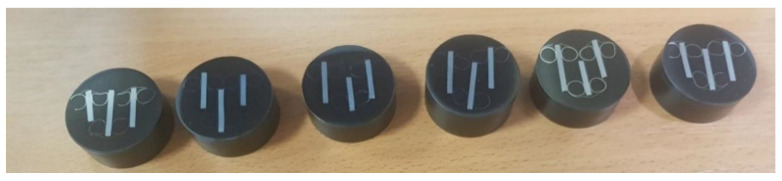
Ready-made samples prepared for testing, embedded in phenolic resin.

**Figure 23:**
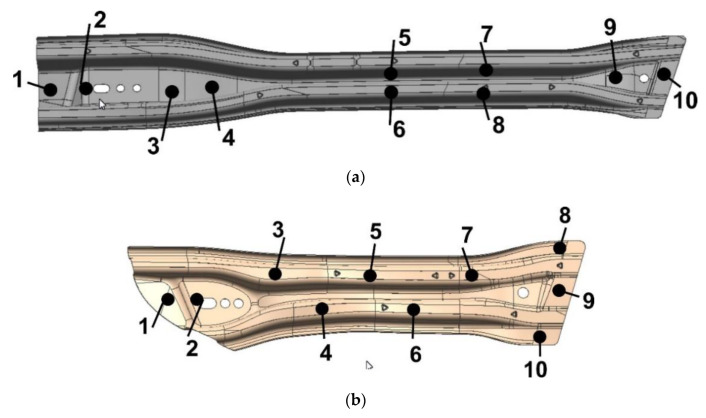
Measuring points when assessing strength properties after pressing (**a**) long beam (**b**) short beam.

In the next stage, destructive tests were carried out. Samples were cut from ready-made beams in selected places and prepared for microstructure tests (Figure 22). These samples were cut around points 1, 4, and 10 for the long beam and 1, 4, and 9 for the short beam (Figure 23).

For these samples, hardness was tested according to the Vickers scale with the use of the HVD-50AP Vickers Hardness Tester and the expected hardness values in the HV = 400 ÷ 550 range were confirmed. The sample results are shown in Table 1.

**Figure 24:**
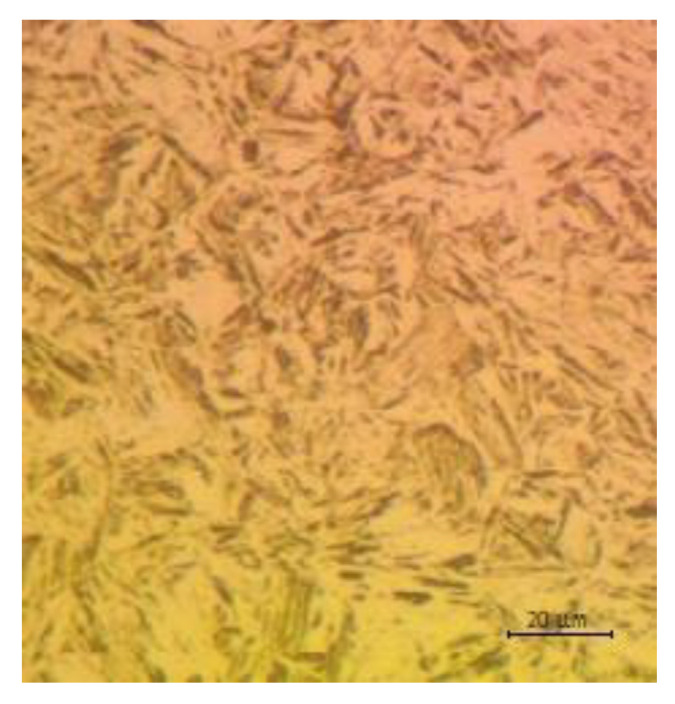
Structure of the sample taken from the first point of the long beam.

Each of the samples was analyzed using a specialized microscope AmScope ME1200TC-5M. Figure 24 is a photo from the microscope image, which shows the structure of the sample taken from the measuring point 1 of the long beam. This is a desirable martensitic structure. This structure was observed for all samples taken at designated measuring points.

**Figure 25:**
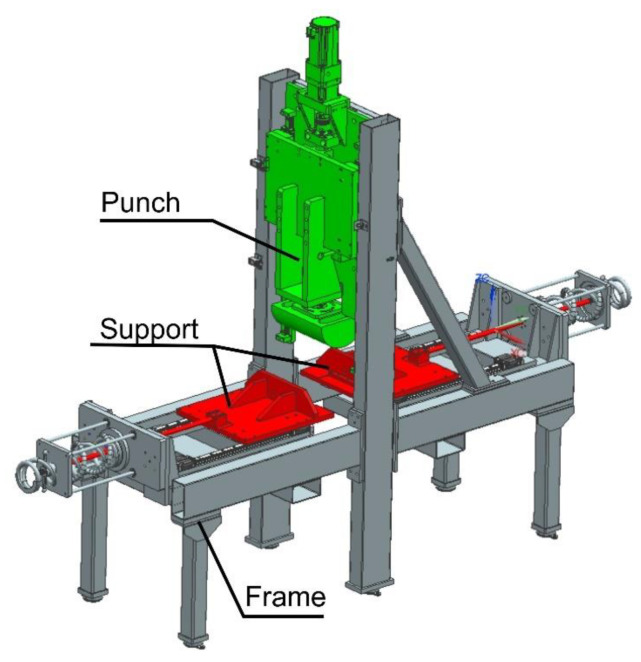
Stand for three-point bending of door beams.

**Figure 26:**
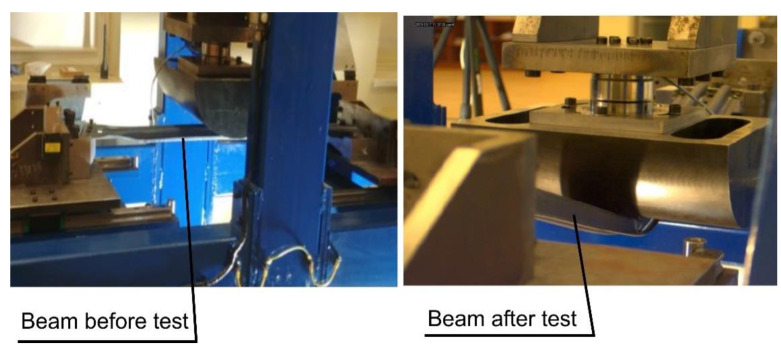
Photos of the stand for three-point bending of beams during the test.

**Figure 27:**
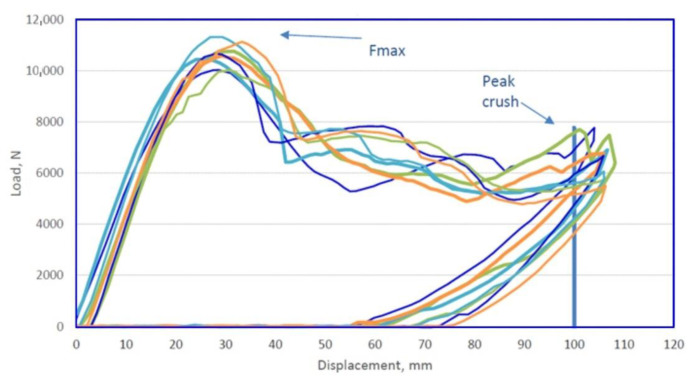
Value of the punch force as a function of deflection for the short beam.

**Figure 28:**
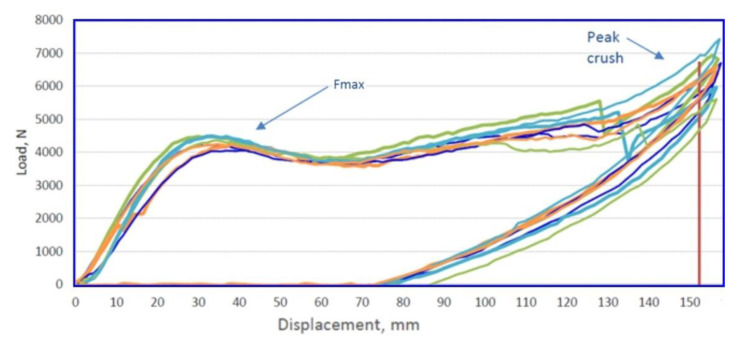
Value of the punch force as a function of deflection for the long beam.

The last test that finished products are subjected to is the three-point bending test, carried out in accordance with the requirements of the customer. The test is performed on a specially designed stand (Figure 25). Its main elements are the supports, in which the beams are fixed, and the half-cylindrical sliding punch. During such an experiment (Figure 26), the force on the punch is measured, with the punch stroke changing from zero to the set value (over 100 mm for the short beam and over 152 mm for the long beam). For the short beam, according to the customer’s requirements, the force Fmax (see Figure 27 and Figure 28) on the punch should be greater than 8 kN, and for the long beam, this value is 3.5 kN.

The tests were carried out for 10 pieces of each type of beams. The figure below presents graphs showing the value of the punch force as a function of the beam deflection, for the short beam (Figure 27) and the long beam (Figure 28). By analyzing the test results, it was found that the beams meet the set requirements. The measured forces exceed the required values.

## 6. Summary and Conclusions

The paper proposes an innovative, two-stage approach to solving the problem of selecting the tool cooling system for hot stamping. The first stage uses functionality of commercial software for simulating hot stamping processes Autoform 7.0., which allows for an approximate but very fast analysis of the tool cooling process. In the second stage, a complete CFD analysis was performed. Temperature distributions on the working surfaces of the tools obtained by analyzing the simplified model and thermal-flow analyses are similar. The temperature values on the tool surfaces recorded at the end of the process obtained by analyzing the simplified model are higher than those obtained by the CFD analysis. In some points, the difference is around 40 °C. This discrepancy can be explained by approximations that are adopted during the analyses in the Autoform package. First, Autoform does not take into account the flow of the cooling medium through the tool, but merely the heat exchange between the cooling channels and the working surfaces of the tools. Nevertheless, such an analysis, due to its short preparation time, is always performed at the tool design stage, especially as it gives a good estimate of the results in advance. In fact, it turns out that the temperature values at the end of the process are slightly lower, which is more favorable for the drawpiece hardening process.

As already mentioned, the advantage of simulations made with the Autoform software is the short preparation time of the calculation model and the short time of the analysis. For a sample die and punch, the development time for the calculation model and simulation in Autoform is about 8 h. In turn, the development time of the models and simulation time for the same die and punch in SimCenter is approximately 80 h. It must be remembered that in the latter case, two tasks must be formulated and solved. The die heating and cooling process and the punch heating and cooling process are analyzed separately (these are two different models). The complexity of both of these tasks may also be demonstrated by the amount of disk space used by CAD models, discrete models and files with analysis results. They took up 160 GB of memory on the disk for the analyzed models. Similar models and results of simplified analyses of tool cooling in Autoform used up 9.5 GB of memory on the disk.

The disadvantages of rough analyses include limitations related to the shape of the cooling channels–these can only be channels with straight axes and a circular cross-sectional shape. This makes it impossible to analyze tools with conformal channels made with additive technologies. In addition, since these simulations do not solve the fluid flow problem, it is more difficult to identify tool overheating points resulting from discontinuities or faulty design.

The tests carried out for the trial series of drawpieces confirmed the correctness of the adopted design assumptions. Both hardness and the mechanical properties were within the required range. A metallographic analysis of the samples showed the formation of the desired martensitic structure in the drawpiece. In turn, the required stiffness of the produced beams was confirmed in the three-point bending test.

## Figures and Tables

**Table 1:** Results of the hardness measurements.

Long Beam		
Measure point	Imprint	Hardness HV
1	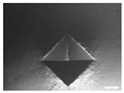	466.4
4	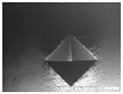	481.6
10	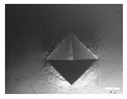	477.2
**Short Beam**		
Measure point	Imprint	Hardness HV
1	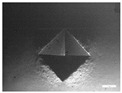	454.9
4	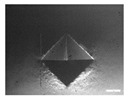	464.2
9	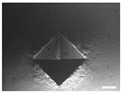	453.9

## Data Availability

Not applicable.
